# Efficacy and Safety of WCFA19 (*Weissella confusa* WIKIM51) in Reducing Body Fat in Overweight and Obese Adults

**DOI:** 10.3390/jcm13092559

**Published:** 2024-04-26

**Authors:** Hwayeon Sun, Jinyoung Shin, Min-ji Kim, Sunghwan Bae, Nicole Dain Lee, Byungwook Yoo

**Affiliations:** 1 Department of Family Medicine, Soonchunhyang University Seoul Hospital, Seoul 04401, Republic of Korea; ssunhwayeon@gmail.com (H.S.);; 2 Department of Family Medicine, Konkuk University Medical Center, Seoul 05030, Republic of Korea; jyshin@kuh.ac.kr; 3 Department of Radiology, Soonchunhyang University Seoul Hospital, Seoul 04401, Republic of Korea; eddybae@schmc.ac.kr; 4 School of Medicine, Georgetown University, Washington, DC 20007, USA; ndl37@georgetown.edu

**Keywords:** probiotics, *Weissella confusa* WIKIM51, *Lactobacillus fermentum*, kimchi, obesity, overweight, anti-obesity

## Abstract

**Background**: WCFA19 (*Weissella confusa* WIKIM51), found during the fermentation of kimchi, is known for its inhibitory effects on body weight and body fat. This study looked at the impact of WCFA19 isolated from dandelion kimchi on weight loss in overweight and obese adults that are otherwise healthy. **Methods**: This study was conducted as a multicenter, double-blind, randomized, placebo-controlled study with 104 overweight and obese subjects. Subjects were randomized evenly into the test group (WCFA19, 500 mg, *n* = 40) or control group (*n* = 34) for 12 weeks from 14 June 2021 to 24 December 2021. Effects were based on DEXA to measure changes in body fat mass and percentage. **Results**: Among the 74 subjects analyzed, WCFA19 oral supplementation for 12 weeks resulted in a significant decrease in body fat mass of 633.38 ± 1396.17 g (*p* = 0.0066) in overweight and obese individuals in the experimental group. The control group showed an increase of 59.10 ± 1120.57 g (*p* = 0.7604), indicating a statistically significant difference between the two groups. There was also a statistically significant difference (*p* = 0.0448) in the change in body fat percentage, with a decrease of 0.41 ± 1.22% (*p* = 0.0424) in the experimental group and an increase of 0.17 ± 1.21% (*p* = 0.4078) in the control group. No significant adverse events were reported. **Conclusions**: Oral supplementation of 500 mg of WCFA19 for 12 weeks is associated with a decrease in body weight, particularly in body fat mass and percentage.

## 1. Introduction

According to the World Health Organization (WHO), a BMI of 25 and higher is considered overweight, and a BMI of 30 and higher is considered obese. As of 2016, over 1.9 billion adults aged 18 and older were overweight, of which, over 650 million were considered obese [1]. The World Obesity Atlas, published on World Obesity Day, predicts that based on current trends, 51% of the world will be overweight or obese by 2035 [2]. Obesity is characterized by an imbalance in which caloric intake exceeds caloric expenditure, which leads to an accumulation of the excess calories as body fat. Obesity increases one’s risk for various diseases including hyperlipidemia, type 2 diabetes, osteoarthritis, hypertension, coronary artery disease, breast cancer, colorectal cancer, and endometrial cancer, and the increased risk of these diseases contributes to higher mortality rates [3].

Korea’s obesity rate has been maintained around 30.0% during the past decade, from 31.7% in 2007 to 34.6% in 2018, and is predicted to increase in the future [4]. With the increase in obesity rates, socio-economic problems associated with obesity are expected to increase, respectively. The WHO reports that globally, obesity-related healthcare costs already account for 2 to 8% of total healthcare costs [5]. The socio-economic losses due to obesity are expected to accelerate further due to other factors including aging [6], making obesity treatment increasingly important [7]. Obesity treatment methods include lifestyle modifications such dietary, exercise, and behavioral therapies, and pharmaceutical and surgical treatments [8]. However, surgical treatments such as endoscopic weight loss procedures like intragastric balloon insertion, endoscopic sleeve gastroplasty, and aspiration therapy, are limited in long-term weight loss maintenance and require further discussion on patient selection for such procedures [9]. Pharmaceutical treatments for obesity include drugs like Orlistat, which inhibits fat absorption, and Lorcaserin and Naltrexone SR/Bupropion SR, which suppress appetite. However, gastrointestinal side effects such as diarrhea, flatulence, fatty stools, digestive disorders, and other side effects like dizziness, headache, and insomnia, have been reported with these medications [10]. Therefore, there has been growing interest in treatments developed from natural substances and functional foods known for their safety and effectiveness. However, due to recent concerns regarding side effects and the use of chemicals in functional food products, there is increased research being conducted on probiotics as a safer alternative treatment for obesity, specifically focusing on the use of live microorganisms that positively influence the host’s health by enhancing the microbial environment of the digestive system [11]. Previous studies suggest that probiotics may play a role in the treatment and prevention of obesity by increasing microbial diversity, inhibiting pathogenic bacterial growth, stimulating short-chain fatty acid production, and regulating appetite [12].

*Weissella confusa* is a lactic acid-producing bacterium that is characteristically similar to *Leuconostoc* spp., is gram-positive, catalase-negative, non-motile, with short rod-shaped firmicutes. It has been isolated from clinical and human fecal samples, as well as from dairy and meat products, and can be used as a secondary starter culture [13,14,15]. In particular, *W. confusa* WIKIM51, isolated from dandelion kimchi, is found in various fermented foods including vegetable, dairy, starch, cereal, meat, and fish-based fermented food [16]. It is abundantly present in the initial fermentation stages of kimchi, a traditional Korean food, and has been proven to be safe as a food ingredient in terms of safety, antibiotic resistance, hemolytic activity, and revertant mutation tests in Korea [17,18,19,20]. Relatedly, studies suggest that the probiotics *W. cibaria* WIKIM28 and *W. confusa* WIKIM51, isolated from kimchi, inhibit the differentiation and lipid accumulation of fat cells and reduce the expression of factors involved in fat cell differentiation [21,22]. Particularly, cell cultures have confirmed the inhibitory effects on lipid accumulation, decreased expression of genes related to fat cell differentiation and synthesis, while animal models have demonstrated anti-obesity effects by inhibiting weight and fat mass gain, reducing dietary intake, decreasing the size of fat cells in the liver and adipose tissue, and reducing obesity-related blood markers.

Therefore, this study aims to evaluate the efficacy and safety of WCFA19 (*W. confusa* WIKIM51) treatment in reducing body fat compared to placebo treatment in overweight and obese individuals.

## 2. Materials and Methods

### 2.1. Study Design

This was a double-blind, placebo-controlled, multicenter study, randomized controlled trial (RCT). The control group was matched with the experimental group at a 1:1 ratio using a block randomization method with allocation codes. The randomization table was generated using the SAS^®^ system’s randomization program, applying a permutation of random numbers (A/B + random number) sequentially to the subject numbers (1 to *n*, the total number of subjects in the trial). The randomization table was pre-designed and created using SAS^®^ (Version 9.4, SAS Institute, Cary, NC, USA) prior to the start of the trial. The sponsor, without researcher involvement, provided the labeled experimental products based on the randomization table, attached to the packaging, to the trial site before the start of the trial. Experimental and control group subjects were provided with randomized and labeled experimental products in the order of their participation in the experiment. Subjects in the experimental group consumed the experimental product WCFA19 (*W. confusa* WIKIM51) at a dose of 500 mg (1 × 10^10^ CFU) once daily with water; subjects in the control group were provided with a visually identical 500 mg placebo capsule. A total of 104 subjects were enrolled, considering a dropout rate of 30%. To monitor compliance, subjects were asked to return every 4 weeks over a 12-week period to report any side effects, return unused capsules, and receive a refill.

The informed consent form obtained approval from the Institutional Review Board of Soonchunhyang University Hospital (Approval No. 2021-01-022), and all patients provided written consent before enrollment. This study is registered with the Clinical Research Information Service (CRIS) under the registration number KCT0007142. The research was conducted in accordance with the protocols and ethical guidelines outlined in the 2000 revision of the Helsinki Declaration and applicable laws.

### 2.2. Study Population

This is a multi-center randomized controlled crossover trial adhering to the CONSORT guidelines. Eligible participants were individuals aged 19 to 65 with a body mass index (BMI) between 25 kg/m^2^ and 32 kg/m^2^ at both visits, 1 and 2. All individuals voluntarily agreed to the informed consent form. Exclusion criteria included the following: (1) Severe cardiovascular, immune, respiratory, hepatobiliary, renal, genitourinary, neurological, musculoskeletal, psychiatric, infectious diseases, and malignancies currently under treatment. (2) Use of contraceptive pills or female hormones within the last 30 days from visit 1. (3) Use of medications affecting body weight (anti-obesity drugs such as appetite suppressants, fat absorption inhibitors, GLP-1 receptor agonists), psychotropic drugs for depression and schizophrenia, beta-blockers, diuretics, steroids, health functional foods/supplements for weight control, and weight control formula foods within the last 2 weeks from visit 1. (4) Antibiotic use within the last 2 weeks from visit 1. (5) Regular consumption (at least four times weekly) of probiotics or continuous use within the last 2 weeks from visit 1. (6) Use of herbal medicine for weight control within the last 3 months from visit 1. (7) Individuals with uncontrolled hypertension (systolic blood pressure ≥ 160 mmHg or diastolic blood pressure ≥ 100 mmHg, measured after 10 min of subject stabilization. (8) Individuals with fasting blood glucose levels of 126 mg/dL or higher or those receiving antidiabetic drugs (oral hypoglycemic agents, insulin, etc.) (9) Thyroid-stimulating hormone (TSH) below 0.1 μIU/mL or above 10 μIU/mL. (10) Creatinine levels exceeding twice the upper normal limit of the test facility. (11) Aspartate aminotransferase (AST or GOT) or alanine aminotransferase (ALT or GPT) levels exceeding three times the upper normal limits. (12) Individuals with severe gastrointestinal disorders that may affect the ingestion of experimental product for the study. (13) Individuals with alcohol-induced disorders and central nervous system disorders. (14) Individuals with musculoskeletal disorders deemed unable to exercise. (15) Individuals who experienced a weight change of 10% or more within the last 3 months from visit 1. (16) Participation in a commercial obesity program within the last 3 months from visit 1. (17) Participation in other interventional clinical trials (including human trials) within the last month or plans to participate in other interventional clinical trials (including human trials) after the start of this trial. (18) Pregnant or breastfeeding individuals or individuals planning to become pregnant during the study period. (19) Sensitivity or allergies to the ingredients of the experimental product. (20) Other reasons where the investigator deemed the subject unsuitable for the study.

### 2.3. Measures of Efficacy

The study consisted of a 12-week treatment phase. Patients that met the selection criteria received the experimental product at approximately the same time each day. Treatment compliance was defined as taking at least 80% of the required dosage.

For the primary efficacy assessment, the changes in body fat mass and percentage before and after consumption of the drug were evaluated using DEXA. The secondary efficacy assessments included changes in body weight, waist circumference, hip circumference, waist/hip ratio, body mass index (BMI), lean body mass measured by DEXA, visceral fat area measured by CT, subcutaneous fat area, total abdominal fat area, and visceral/subcutaneous fat area ratio. Blood lipids (Total Cholesterol, HDL, LDL-Cholesterol, Triglyceride), hs-CRP, Adiponectin, Leptin were evaluated with blood samples with the exzymatic colorimetric method at Soonchunhyang University Hospital Seoul and Konkuk University Medical Center Seoul. All measurements were taken in a fasting state. Safety was assessed by analyzing all reported treatment-emergent adverse events (TEAEs), which were defined as any adverse events that first occurred or worsened after the patient was assigned to treatment.

Height, weight, blood pressure, and pulse rate were examined. Body mass index (BMI) was calculated as weight divided by height squared. Demographic data (age, birth date, and sex), comorbidities diagnosed by medical doctors, surgical history within six months, and medication type were collected. Lifestyle data, such as physical activity (none/1–2/3–4/≥5 days per week), smoking status (non-smoker, ex-smoker, current smoker), and alcohol consumption (more or less than once a month) were collected through the Global Physical Activity Questionnaire (GPAQ). Subjects were educated on how to complete dietary survey forms, which were used to collect data on their dietary habits (regular meal: yes/no, mealtime: <10/10–20/≥20 min, and frequency of overeating per week). At visits 2, 3, and 4, the investigators reviewed the completed dietary survey forms, through which the subjects’ daily energy intake was assessed. Dietary and physical activity education was provided to the subject at every visit.

### 2.4. Statistical Analysis

All data are presented as mean ± standard deviation, and statistical significance was assumed at *p* < 0.05. Statistical comparisons of body fat mass, body fat percentage, body weight, waist circumference, hip circumference, waist/hip ratio, body mass index (BMI), lean body mass measured by DEXA, visceral fat area measured by CT, subcutaneous fat area, total abdominal fat area, visceral/subcutaneous fat area ratio, blood lipids (Total Cholesterol, HDL-Cholesterol, LDL-Cholesterol, Triglyceride), hs-CRP, Adiponectin, Leptin before and after the use of the experimental drug were performed using paired *t*-tests or Wilcoxon rank-sum tests. Analyses were conducted using the statistical SAS^®^ software (Version 9.4, SAS Institute, Cary, NC, USA). A per-protocol analysis set (PPS) was used to analyze the clinical evaluation data of the subjects who fully completed the trial with better compliance. A safety set (SS) was used to analyze the safety data of the subjects who took the trial medicine at least once.

## 3. Results

### 3.1. Study Participant Characteristics

Overall, a total of 74 participants were included in this study. The experimental group included 40 individuals, with an average age of 46.33 ± 12.30 (BMI of 27.17 ± 1.66 kg/m^2^, SBP of 127.43 ± 11.61 mmHg, DBP of 76.65 ± 8.61 mmHg, pulse of 76.37 ± 9.49), while the control group included 34 individuals with an average age of 39.76 ± 11.80 (BMI of 26.87 ± 1.77 kg/m^2^, SBP of 128.57 ± 10.08 mmHg, DBP of 78.53 ± 8.59 mmHg, pulse of 79.18 ± 10.48) (Table 1). All participants met the inclusion and exclusion criteria and were considered overweight or obese. The mean compliance was 99.19 ± 5.40% in the experimental group and 99.83 ± 3.01% in the control group, with no statistically significant difference in consumption between the two groups (*p* = 0.5167).

### 3.2. Primary Measure of Efficacy

In the analysis of changes in body fat mass measured by DEXA, the experimental group exhibited a significant decrease of 633.38 ± 1396.17 g (*p* = 0.0066) after 12 weeks of consumption, while the control group exhibited an increase of 59.10 ± 1120.57 g (*p* = 0.7604). This difference between the two groups (WCFA19 and Placebo) was statistically significant (*p* = 0.0229). In the analysis of changes in body fat percentage, the experimental group showed a significant reduction of 0.41 ± 1.22% (*p* = 0.0424) after 12 weeks of consumption, while the control group showed an increase of 0.17 ± 1.21% (*p* = 0.4078). The difference between the two groups was statistically significant (*p* = 0.0448) (Table 2).

### 3.3. Secondary Measure of Efficacy, Laboratory Tests

In terms of changes in weight, the experimental group exhibited a significant decrease of 0.96 ± 1.89 kg (*p* = 0.0027) over the 12 weeks, while the control group exhibited an increase of 0.00 ± 1.32 kg (*p* = 0.9897). The difference between the two groups was statistically significant (*p* = 0.0125). In terms of BMI, the experimental group showed a significant reduction of 0.34 ± 0.68 kg/m^2^ (*p* = 0.0032) over the 12 weeks, while the control group showed an increase of 0.00 ± 0.46 kg/m^2^ (*p* = 0.9703). The difference between the two groups was statistically significant (*p* = 0.0386).

For the changes in waist and hip circumference, the experimental group showed a statistically significant decrease (*p* < 0.0001), and the control group also exhibited a decrease (*p* = 0.0011, 0.0033), but there was no statistically significant difference between the consumption groups.

For the changes in waist-to-hip ratio, after 12 weeks of consumption, the experimental group showed a decrease of 0.01 ± 0.05 (*p* = 0.2099), and the control group showed an increase of 0.01 ± 0.04 (*p* = 0.4224). However, there was no statistically significant difference between the two groups (Table 3).

For the changes in fat-free mass measured by DEXA after 12 weeks of consumption, the experimental group exhibited a decrease of 252.75 ± 1015.85 g (*p* = 0.1237), and the control group exhibited a decrease of 74.31 ± 1006.00 g (*p* = 0.6695). However, there was no statistically significant difference between the two groups.

For the changes in visceral fat area seen in the CT scans after 12 weeks of consumption, the experimental group showed a decrease of 3.07 ± 22.37 cm^2^ (*p* = 0.3900), and the control group showed a decrease of 2.16 ± 20.15 cm^2^ (*p* = 0.5366). However, there was no statistically significant difference between the consumption groups.

In terms of changes in subcutaneous fat area after 12 weeks of consumption, the experimental group showed a decrease of 4.16 ± 26.42 cm^2^ (*p* = 0.3256), and the control group showed an increase of 5.78 ± 21.55 cm^2^ (*p* = 0.1273). However, there was no statistically significant difference between the consumption groups.

In terms of changes in the visceral/subcutaneous fat area ratio after 12 weeks of consumption, the experimental group showed an increase of 0.01 ± 0.17 (*p* = 0.8566), and the control group exhibited a decrease of 0.02 ± 0.12 (*p* = 0.3344). However, there was no statistically significant difference between the consumption groups.

In terms of changes in total cholesterol levels, the experimental group showed in an increase after 12 weeks of consumption while the control exhibited a decrease, but there was no statistically significant difference between the two groups (*p* = 0.174) (Table 4). For HDL-Cholesterol, there was no statistically significant difference in levels between the two groups (*p* = 0.96). LDL-Cholesterol levels increased in the experimental group and decreased in the control group, but there was no statistically significant difference between the two groups (*p* = 0.53). For changes in hs-CRP, the experimental group showed a decrease of 0.03 ± 1.00 mg/L (*p* = 0.8487) after 12 weeks, and the control group showed an increase of 0.24 ± 1.79 mg/L (*p* = 0.4330), but there was no statistically significant difference between the two groups. 

In the analysis of changes in adiponectin levels after 12 weeks of consumption, the experimental group demonstrated a decrease of 0.82 ± 1.89 μg/mL (*p* = 0.0089), and the control group showed a decrease of 0.90 ± 1.52 μg/mL (*p* = 0.0016). However, there was no statistically significant difference between the two groups. For the changes in leptin levels after 12 weeks of consumption, the experimental group exhibited a decrease of 1.39 ± 4.56 ng/mL (*p* = 0.0605), and the control group showed a decrease of 0.58 ± 4.02 ng/mL (*p* = 0.4075). There was no statistically significant difference between the two groups.

Analyzing the changes in leptin levels with the PP Set, after 12 weeks of consumption, the experimental group demonstrated a decrease of 1.39 ± 4.56 ng/mL (*p* = 0.0605), and the control group showed a decrease of 0.58 ± 4.02 ng/mL (*p* = 0.4075). There was no statistically significant difference between the two groups.

There were no significant changes in hematologic or blood chemistry parameters except for AST and ALP changes in the WCFA19 group (Table 5) using the safety set (experimental group *n* = 51, control group *n* = 51). However, there were no subjects identified by the investigators where these changes were deemed clinically significant. Other adverse reactions, such as blood pressure and EKG, were monitored during the course of the study.

### 3.4. Adverse Events

Adverse reactions were compared using the safety set (test group *n* = 51, control group *n* = 51). In all, 9 individuals (17.65%) experienced adverse reactions in the experimental group, while 11 individuals (21.57%) reported adverse reactions in the control group. Adverse reactions confirmed to be related to the test drug included two cases in the test group (lower abdominal discomfort, waist pain) and one case in the control group (rash on arms/legs, tingling). As a result, one participant discontinued the trial prematurely, and completely recovered from the adverse reactions. Apart from these instances, no significant adverse reactions related to this clinical trial were reported.

## 4. Discussion

This study investigated the effects of the dandelion kimchi-derived probiotic WCFA19 in weight loss in overweight and obese adults with a BMI between 25 kg/m^2^ and 32 kg/m^2^ over a 12-week consumption period. 

After WCFA19 consumption, the experimental group showed a significant decrease in weight (*p* = 0.0027), while the control group exhibited an increase (*p* = 0.9897), indicating a statistically significant difference between the groups (*p* = 0.0125). DEXA results also revealed a decrease in both body fat mass and percentage in the experimental group, whereas the control group showed an increase (*p* = 0.0229, 0.0448). This study not only focused on basic weight changes but also confirmed significant alterations in body fat mass and percentage through DEXA measurements. The absence of a significant decrease in muscle mass, despite weight loss in the experimental group, suggests a meaningful change. Additionally, limitations in evaluating changes in subcutaneous fat area were acknowledged due to the method’s sectional nature.

Although obesity is a common health issue in our current society, its various complications such as metabolic syndrome, hypertension, heart disease, cancer, and sleep disorders make obesity a serious health issue to consider. However, treating obesity with drug therapy remains problematic due to various side effects, emphasizing the need for safer and more effective drug discoveries and treatment development.

In this study, we confirmed significant changes not only in basic weight measurement but also in body fat mass and percentage through DEXA measurements. Specifically, the meaningful decrease in body weight without a significant reduction in muscle mass in the experimental group is noteworthy. We are able to accurately measure the amount of body fat mass through DEXA measurements. Although BMI is commonly used to measure obesity, it does not consider the crucial aspects of fat distribution that DEXA measurements do [23]. Although the ratio of body weight to body fat mass is a vital measure of health and fitness as it includes bones, organs, muscles, and body fat, it is limited as a useful indicator as it does not consider body fat and muscle mass. Therefore, DEXA measurements that provide body fat mass and body fat percentage measurements are a more accurate assessment of the amount of fat relative to overall weight. This allows for a more comprehensive evaluation of the risks of diseases such as heart disease, diabetes, and specific types of cancer. Thus, while body weight is an important measure of health and fitness, body fat mass and body fat percentage can be considered more crucial indicators of overall health [24]. Therefore, many researchers use DEXA measurements as a valid evaluation criterion for weight loss studies, like this study.

In recent years, many studies have highlighted the potential connection between an imbalance of gut microbiota and the development of obesity [25,26]. Healthy gut microbiota is essential for maintaining the body’s metabolism and balance in energy. An imbalance in gut microbiota can lead to metabolic disorders, an increase in central appetite, and potentially, obesity [27]. Relatedly, studies have demonstrated that transplanting the gut microbiota from an obese subject into germ-free mice can transfer the obese phenotype. These findings suggest that gut microbiota play a role in obesity and may be a potential target for prevention and treatment of metabolic disorders like long-term obesity [28]. Ultimately, the regulation of gut microbiota could be considered a non-toxic and safe potential therapeutic approach for treating metabolic disorders associated with obesity. 

Although the exact mechanisms through which gut microbiota contribute to human health are still unclear, gut microbiota affect the stimulation of the immune system, improvement of digestion and absorption, synthesis of vitamins, and reduce gas production [29,30]. Particularly, gut microbiota and its metabolites have a significant influence on host metabolism, explaining various characteristics of obesity and metabolic syndrome (MS) [31,32].

A literature review on the modulation of gut microbiota by prebiotics indicates that *Bifidobacterium* spp. plays a crucial role in improving obesity by promoting its growth in the presence of prebiotics [33]. However, research suggests that the stimulatory effects of prebiotics are not limited to *Bifidobacterium* spp., *Lactobacillus* spp., and *Faecalibacterium prausnitzii* as they also affect other bacterial taxa [34,35]. In particular, prebiotic-induced changes in gut microbiota composition indirectly influence various pathological aspects associated with obesity, ultimately leading to weight loss. These findings from many studies demonstrate that probiotics safely contribute to weight loss.

Kim et al. studied the effects of consuming probiotics containing *Lactobacillus gasseri* BNR17 for 12 weeks on the body composition of obese women aged 40–60 using DEXA [36]. The study demonstrated a significant reduction in body fat mass in the experimental group that consumed the probiotic strain. Similarly, Cho et al. studied the effects of probiotics containing *L. curvatus* HY7601 and *L. plantarum* KY1032 for 12 weeks on the body composition of adult men using DEXA. The study reported a significant decrease in abdominal fat and total body fat mass in the experimental group that consumed the probiotics [37], which aligns with the findings of this current study. 

In a meta-analysis reviewing probiotics, out of 14 randomized controlled trials (RCTs), 9 studies reported weight loss and body fat reduction, 3 studies reported no significant effects, and 2 studies reported weight gain. Out of 33 RCTs, although 22 studies reported the occurrence of side effects, and these were temporary side effects including constipation, diarrhea, and nausea [38]. 

Specifically, within RCT studies related to *Lactobacillus* spp., Sohn’s 2013 study reported loss in weight, visceral fat area, and subcutaneous fat area, and a decrease in BMI after 12 weeks of *Lactobacillus* spp. consumption [39]. Sanchez et al. reported a loss in body weight and body fat mass in obese men and women after consumption of probiotics containing *L. rhamnosus* CGMCC1.3724 [40], which supports the findings of this study. However, Jung’s 2013 study on *L. gasseri* BNR17 showed a decrease in weight in the experimental group but no significant difference compared to the placebo group [41]. Similarly, Kadooka’s 2013 study on *L. gasseri* SBT2055 showed a decrease in BMI and waist and hip circumference during the first four weeks, but the reduction diminished by 12 weeks [42]. 

WCFA19 contains *W. confusa* WIKIM51, a probiotic isolated from dandelion kimchi. Cell cultures confirm the inhibitory effects of *W. confusa* WIKIM51 on lipid accumulation and decreased expression of genes related to adipocyte differentiation, and animal models demonstrate its anti-obesity effects including the suppression of increase in body weight and body fat, reduced dietary intake, decreased size of fat cells in the liver and adipose tissue, and a decrease in obesity-related blood markers [43]. Lim’s study evaluated the efficacy of kimchi-derived probiotic *W. confusa* WIKIM51 on improving obesity in both in vitro and in vivo settings. They found that WIKIM51 inhibited the differentiation of fat cells in 3T3-L1 cells induced for adipocyte differentiation. This inhibition was associated with the suppression of key genes related to lipid metabolism including Pparγ, C/EBPα, Srebp-1c, and Fas. WIKIM51 also increased the expression of genes associated with energy expenditure including Pparα and Cpt. Through these regulatory mechanisms, WIKIM51 effectively suppresses the formation of fat cells, leading to weight loss [43].

Additionally, modulation of the gut microbiome through supplementation with probiotics has been shown to reduce low-grade intestinal inflammation and improve the integrity of the intestinal barrier, promoting homeostatic–metabolic balance [44]. Therefore, using these microorganisms, WIKIM51 as probiotics can be expected to reduce chronic inflammation in the intestines, weaken insulin resistance, prevent obesity-related metabolic disorders, and reduce body fat accumulation [45].

There are a few limitations to this study including the relatively short duration, small sample size, and lack of dose-response data. The enrollment and evaluation of subjects using a self-reported questionnaire might have introduced bias into the study findings. 

Firstly, during the course of this study, external factors such as holidays (Thanksgiving in the United States, Chinese New Year, and traditional Korean holidays) could have led to a decrease in physical activity and an increase in caloric intake. The inability to control such external factors, including the difficulty subjects faced in regulating their diet during these personal vacations, made it challenging to maintain uniform caloric intake. For this reason, it was unavoidable to report a limited effect size. 

Secondly, this study did not investigate the duration of the effects of *W. confusa* after the discontinuation of probiotic consumption. Further research is necessary to address this gap. 

Thirdly, probiotics are considered living organisms, and thus cannot be treated as conventional drugs in trials. Unlike drugs, where consistent results can be expected with standard storage and consumption practices, probiotic strains may yield varied results depending on factors including storage methods and consumption practices. Many papers suggested that the effects of probiotics were limited to the window of supplementation (Perspective: fundamental limitations of the randomized controlled trial method in nutritional research: the example of probiotics).

Lastly, while this study looked at biochemical variables such as inflammatory biomarkers and blood chemistry, it did not assess changes in gut microbiota before and after *W. confusa* consumption.

## 5. Conclusions

This study conducted a randomized controlled trial to investigate the effects of kimchi-derived probiotic WCFA19 on body fat reduction in healthy overweight individuals over 12 weeks of consumption. The results of the study suggest that kimchi-derived probiotics have an effect on reducing body weight and body fat mass. Subsequent follow-up studies examining the impact and mechanism of kimchi-derived probiotics on gut microbiota could contribute to finding medical applications for kimchi-derived probiotics as functional foods for overweight or obese individuals or individuals with related metabolic disorders.

## Figures and Tables

**Table 1:** Characteristics of studies.

	WCFA19 (N = 40)	Placebo (N = 34)	*p*-Value
Gender (Male)	17 (42.5)	17 (50)	
Gender (Female)	23 (57.5)	17 (50)	
Age (years)	46.33 ± 12.30	39.76 ± 11.80	0.0227 *
Height (cm)	164.21 ± 9.21	167.19 ± 10.07	0.1879
Weight (kg)	73.55 ± 9.78	75.44 ± 10.72	0.4304
BMI (kg/m^2^)	27.17 ± 1.66	26.87 ± 1.77	0.2875
Waist measure (cm)	93.09 ± 6.71	91.22 ± 6.72	0.2360
SBP (mmHg)	127.43 ± 11.61	128.57 ± 10.08	0.5985
DBP (mmHg)	76.65 ± 8.61	78.53 ± 8.59	0.2719
Pulse	76.37 ± 9.49	79.18 ± 10.48	0.1599

N (%), Mean ± SD. BMI: Body mass index, SBP: Systolic blood pressure, DBP: Diastolic blood pressure. * *p* < 0.005; Significant compared to the WCFA19 group and the Placebo group.

**Table 2:** Primary measure of efficacy: Change of DEXA measures before and after intervention with WCFA19 or placebo (PP Set).

Parameter	WCFA19 (*n* = 40)		Placebo (*n* = 34)		*p* ^1^
	Change from Baseline	*p*-Value	Change from Baseline	*p*-Value	
Body Fat Mass	−633.38 ± 1396.17	0.0066 **	59.10 ± 1120.57	0.7604	0.0229 *
Percent Body Fat (%)	−0.41 ± 1.22	0.0424 *	0.17 ± 1.21	0.4078	0.0448 *

*p* ^1^: Compared between WCFA19 and placebo group. * *p* < 0.05. ** *p* < 0.001.

**Table 3:** Changes in secondary measure of efficacy (PP Set).

Parameter	WCFA19 (*n* = 40)		Placebo (*n* = 34)		*p* ^1^
	Change from Baseline	*p*-Value	Change from Baseline	*p*-Value	
Weight (kg)	−0.96 ± 1.89	0.0027 **	0.00 ± 1.32	0.9897	0.0125 (T) *
BMI	−0.34 ± 0.68	0.0032 **	0.00 ± 0.46	0.9703	0.0386 (W) *
Waist circumference (cm)	−3.68± 4.32	<0.0001 ***	−2.05 ± 3.34	0.0011 **	0.0787 (T)
Hip circumference (cm)	−2.76 ± 3.15	<0.0001 ***	−2.06 ± 3.78	0.0033 **	0.3871 (T)
Waist/hip ratio	−0.01 ± 0.05	0.2099	0.01 ± 0.04	0.4224	0.1451 (W)
Body fat mass (g)	−252.75 ± 1015.85	0.1237	−74.31 ± 1006.00	0.6695	0.4519 (T)
Visceral fat area (cm^2^)	−3.07 ± 22.37	0.39	−2.16 ± 20.15	0.5366	0.8547 (T)
Subcutaneous fat area	−4.16 ± 26.42	0.3256	5.78 ± 21.55	0.1273	0.0839 (T)
Total abdominal fat area	−7.23 ± 27.17	0.1002	3.62 ± 26.38	0.4288	0.0868 (T)
Visceral/subcutaneous fat area ratio	0.01 ± 0.17	0.8566	−0.02 ± 0.12	0.3344	0.9144 (W)

*p*-value: Compared between groups; *p*-value for Two sample *t*-test (T) or Wilcoxon rank sum test (W). *p* ^1^: Compared between WCFA19 and placebo group. * *p* < 0.05. ** *p* < 0.001. *** *p* < 0.0001.

**Table 4:** Changes in secondary measure of efficacy between WCFA19 and placebo groups at baseline (week 0) and after intervention (week 12).

	Parameter	WCFA19 (*n* = 40)		Placebo (*n* = 34)		*p* ^1^
		Change from Baseline	*p*-Value	Change from Baseline	*p*-Value	
	Total Cholesterol (mg/dL)	5.28 ± 29.52	0.2652	−2.15 ± 23.82	0.6027	0.2435 (T)
Lipid	HDL-Cholesterol (mg/dL)	1.70 ± 6.87	0.1256	1.76 ± 5.52	0.0713	0.9649 (T)
	LDL-Cholesterol (mg/dL)	2.13 ± 28.10	0.6351	−1.35 ± 19.24	0.6844	0.5318 (T)
	Triglyceride (mg/dL)	−5.73 ± 44.19	0.4176	−18.03 ± 103.13	0.3154	0.7285 (W)
	hs-CRP (mg/L)	−0.03 ± 1.00	0.8487	0.24 ± 1.79	0.4330	0.9956 (W)
	Adiponectin (μg/mL)	−0.82 ± 1.89	0.0089 **	−0.90± 1.52	0.0016 **	0.7490 (W)
	Leptin (ng/mL)	−1.39 ± 4.56	0.0605	−0.58 ± 4.02	0.4075	0.4216 (T)

*p*-value: Compared between groups; *p*-value for Two sample *t*-test (T) or Wilcoxon rank sum test (W). *p* ^1^: Compared between WCFA19 and placebo group. ** *p* < 0.001.

**Table 5:** Change of blood biochemistry between WCFA19 and placebo groups at baseline (week 0) and after intervention (week 12) (safety set).

Parameter	WCFA19 (*n* = 51)		Placebo (*n* = 51)		*p* ^1^
	Change from Baseline	*p*-Value	Change from Baseline	*p*-Value	
WBC (10^3^/μL)	−0.11 ± 1.07	0.4752	0.04 ± 0.98	0.7612	0.4656 (T)
RBC (10^6^/μL)	0.01 ± 0.20	0.7405	−0.00 ± 0.19	0.9318	0.7657 (T)
Hb (g/dL)	0.05 ± 0.72	0.6601	−0.05 ± 0.54	0.5307	0.4667 (T)
Hct (%)	0.20 ± 2.02	0.5089	0.07 ± 1.75	0.7994	0.7441 (T)
MCV (fL)	0.20 ± 2.32	0.5608	0.19 ± 1.65	0.4357	0.7725 (W)
Platelet(10^3^/μL)	−5.13 ± 34.96	0.3200	4.69 ± 21.75	0.1553	0.1084 (T)
Neutrophil (%)	−1.77 ± 7.13	0.0953	−1.46 ± 6.15	0.1190	0.8217 (T)
Lymphocyte (%)	1.82 ± 6.61	0.0652	1.50 ± 5.83	0.0921	0.8035 (T)
Monocyte (%)	0.34 ± 0.99	0.0236 *	−0.07 ± 1.16	0.6922	0.0730 (T)
Eosinophil (%)	−0.39 ± 1.95	0.1790	−0.03 ± 0.98	0.8218	0.9377 (W)
Basophil (%)	0.00 ± 0.25	0.9861	0.06 ± 0.25	0.0992	0.2383(T)
Glucose (mg/dL)	−1.09 ± 7.91	0.3517	0.16 ± 8.63	0.9044	0.4738 (T)
Protein (g/dL)	0.01 ± 0.36	0.8719	−0.00 ± 0.37	0.9680	0.8881 (T)
Albumin (g/dL)	−0.04 ± 0.21	0.2414	−0.01 ± 0.21	0.7219	0.5659 (T)
Total bilirubin (mg/dL)	−0.00 ± 0.25	0.9765	0.03 ± 0.24	0.4274	0.5564 (T)
AST (IU/L)	1.51 ± 4.65	0.0308 *	−1.82 ± 12.57	0.3361	0.0031 (W) **
ALT (IU/L)	1.04 ± 10.09	0.4824	−1.93 ± 16.08	0.4243	0.2137 (T)
ALP (IU/L)	2.02 ± 5.36	0.0130 *	1.29 ± 9.88	0.3861	0.2534 (W)
BUN (mg/dL)	0.47 ± 2.70	0.2385	−0.06 ± 3.37	0.9090	0.4081 (T)
Creatinine (mg/dL)	0.00 ± 0.07	0.6870	0.00 ± 0.07	0.7231	0.9745 (T)
Ca (mg/dL)	0.09 ± 0.40	0.1509	0.06 ± 0.41	0.3669	0.7266 (T)
Na (mmol/L)	−0.28 ± 2.38	0.4304	−0.11 ± 2.61	0.7768	0.7516 (T)
K (mmol/L)	0.04 ± 0.36	0.4721	0.02 ± 0.38	0.7873	0.7708 (T)
Cl (mmol/L)	−0.26 ± 2.35	0.4609	0.09 ± 2.31	0.7979	0.4815 (T)
Uric acid (mg/dL)	0.05 ± 0.63	0.6145	−0.16 ± 0.84	0.2228	0.1952 (T)
CK (U/L)	9.70 ± 62.89	0.2958	−9.84 ± 42.65	0.1287	0.0172 (W)
γ-GTP(IU/L)	−0.94 ± 6.16	0.3031	−0.53 ± 16.57	0.8300	0.7244 (W)

*p*-value: Compared between groups; *p*-value for Two sample *t*-test (T) or Wilcoxon rank sum test (W). *p* ^1^: Compared between WCFA19 and placebo group. * *p* < 0.05. ** *p* < 0.001.

## Data Availability

For data supporting the reported results, contact the corresponding author.
